# Supplementary material for: Cellular Uptake of a Fluorescent Ligand Reveals Ghrelin O-Acyltransferase Interacts with Extracellular Peptides and Exhibits Unexpected Localization for a Secretory Pathway Enzyme
Source: ACS Chem Biol. 2023 Jul 26;18(8):1880–90. doi: 10.1021/acschembio.3c00334 (PMC10442857; doi:10.1021/acschembio.3c00334)
Supplement: Supplementary file 1 — cb3c00334_si_001.pdf [file cb3c00334_si_001.pdf]

Supporting Information

Cellular uptake of a fluorescent ligand reveals ghrelin *O*-acyltransferase interacts with extracellular peptides and exhibits unexpected localization for a secretory pathway enzyme

Maria B. Campaña,[1,#] Tasha R. Davis,[1,#] Sadie X. Novak,[1] Elizabeth R. Cleverdon,[1] Michael Bates,[2] Nikhila Krishnan,[2] Erin R. Curtis,[2] Marina D. Childs,[3] Mariah R. Pierce,[1] Yasandra Morales-Rodriguez,[1] Michelle A. Sieburg,[1] Heidi Hehnly,[2,4] Leonard G. Luyt,[3,5] and James L. Hougland[1,2,4,*]

[1]Department of Chemistry, Syracuse University, Syracuse, NY 13244, USA
[2]Department of Biology, Syracuse University, Syracuse, NY 13244, USA
[3]Department of Chemistry, University of Western Ontario, London, Ontario, Canada N6A 2K7
[4]BioInspired Syracuse, Syracuse University, Syracuse, NY 13244, USA
[5]Department of Oncology, and Department of Medical Imaging, London Regional Cancer Program, Lawson Health Research Institute, 800 Commissioners Road East, London, Ontario, Canada N6A 5W9

[#] These authors contributed equally to this work

*To whom correspondence should be addressed:

James L. Hougland
Departments of Chemistry and Biology
Syracuse University
1-133 Center for Science and Technology
Syracuse, NY, 13244, USA
Tel: (315)-443-1134
Fax: (315)443-4070
Email: hougland@syr.edu

Supporting Methods

Table S1. Analytical data for peptide ligands

Table S2. Cell imaging and scoring for GOAT transfection and ligand **15** uptake.

Figure S1. Dose-response curves for hGOAT inhibition by peptide ligands

Figure S2. GHSR binding competition assays for peptide ligands

Figure S3. Western blot verification of hGOAT expression in transfected HEK 293 cells.

Figure S4. Western blot verification of anti-MBOAT4 antibody

Figure S5. Immunofluorescence validation of anti-MBOAT4 antibody in HEK293, 22Rv1, and LNCaP cells.

Figure S6. Loss of GOAT immunofluorescence upon peptide competition verifies epitope specificity of anti-MBOAT4 antibody.

Supporting Methods

Peptide synthesis and characterization. Peptide synthesis was carried out using standard Fmoc solid-phase peptide synthesis on a Biotage SyroWave automated peptide synthesizer. Peptides were synthesized on a 0.1 mmol scale using Rink amide MBHA resin (0.39 mmol/g). The resin was initially swelled with dichloromethane (DCM), followed by Fmoc deprotection using 2 mL of 40% piperidine in *N*,*N*-dimethylformamide (DMF) for two cycles (3 min, 12 min). Amino acids were coupled with 4 equiv of Fmoc protected amino acid, 4 equiv. of *O*-(6-chlorobenzotriazol-1-yl)-*N*,*N*,*N′*,*N′*-tetramethyluronium hexafluorophosphate (HCTU), and 8 equiv of *N*,*N*-diisopropylethylamine (DIPEA) in *N*-methylpyrrolidinone (NMP). The mixture was added to the resin and vortexed for 40 min. These cycles were repeated until all amino acids were coupled to the resin.

Allyloxycarbonyl deprotection of diaminopropionic acid was performed manually under inert atmospheric N_2 conditions. DCM was dried over sieves for 24 h before adding 4 mL to the resin. An amount of 24 equiv of phenylsilane was then added to the peptide resin and shaken for 5 min. An amount of 0.15 equiv of tetrakis(triphenylphosphine)-palladium(0) was then added and allowed to react for 10 min. If desired, the resulting free amine was then acylated using 3 equiv of the corresponding acid (octanoic acid or 6-fluoro-2-naphthoic acid), 3 equiv of HCTU, and 6 equiv of DIPEA in DMF. The reaction mixture was left to couple overnight.

Full deprotection of the synthesized peptide was performed by adding a 2 mL mixture of 95% trifluoroacetic acid (TFA), 2.5% triisopropylsilane (TIS), and 2.5% water to the resin and allowing it to mix for 5 h. The cleaved peptide was precipitated from solution using ice-cold *tert*-butyl methyl ether (TBME) and centrifuged (3000 rpm, 10 min, 0 ˚C) resulting in a crude peptide pellet. The supernatant was decanted, and the resulting peptide pellet was dissolved in 20%

acetonitrile in water, frozen at −78 °C, and lyophilized to a white crude powder. Purification was performed using preparative HPLC-MS, and purity of the resulting peptides was analyzed using analytical HPLC-MS. These results are summarized in Supporting Table S1, with all compounds determined to have ≥95% purity.

GHSR receptor binding assays. Peptide binding affinity for the ghrelin receptor was determined using a competitive radioligand-displacement binding assay.[1] Assays were performed using GHS-R1a transfected HEK 293 cells as the receptor source and human His[^{125}I]-ghrelin(1−28) (PerkinElmer Inc. NEX388010UC) as the radioligand. Human ghrelin(1−28) was used as a reference to ensure the validity of the results. A suspension of membrane from HEK 293/GHS-R1a cells (100,000 cells per assay tube) were incubated with ghrelin(1−8) peptide analogues (at concentrations of 10^{-6} M, 10^{-7} M, 10^{-8} M, 10^{-9} M, 10^{-10} M, 10^{-11} M, and 10^{-12} M) and His[^{125}I]-ghrelin (15 pM per assay tube) in binding buffer (25 mM HEPES, 5 mM magnesium chloride, 1 mM calcium chloride, 2.5 mM EDTA, and 0.4% BSA, pH 7.4). The resulting suspension was incubated for 20 min under shaking (550 rpm) at 37 °C. Unbound [^{125}I]-ghrelin was removed and the amount of [^{125}I]-ghrelin bound to the membranes was measured by γ counter. IC_{50} values were determined by nonlinear regression analysis to fit a four-parameter dose−response curve using Prism 6 (version 6.0c). All binding assays were performed in triplicate.

hGOAT inhibition assays. Assays were performed using previously reported protocols.[2-3] For each assay, membrane fraction from Sf9 cells expressing hGOAT was thawed on ice and homogenized by passage through an 18-gauge needle 10 times. Assays were performed with 50 µg of membrane protein, as determined by Bradford assay. Membrane fraction was pre-incubated with 1 µM methyl

arachidonyl fluorophosphonate (MAFP) and unlabeled peptide inhibitor or vehicle as indicated in 50 mM HEPES pH 7.0 for 30 minutes at room temperature prior to reaction initiation.[4] All reactions were initiated by the addition of 1.5 μM $GSSFLC_{AcDan}$ and 300 μM octanoyl CoA. Reactions were incubated for 30 minutes at room temperature under foil. All assays were stopped with the addition of 50 μL of 20% acetic acid in isopropanol, and solutions were clarified by protein precipitation with 16.7 μL of 20% trichloroacetic acid, followed by centrifugation (1,000 x *g*, 2 minutes). The supernatant was then analyzed using reverse-phase HPLC with fluorescence detection as previously described.[2-3] Peak integrations for both substrate and product peaks were calculated using Chemstation for LC (Agilent Technologies). Data reported are the average of three independent determinations, with error reported as standard deviation.

For determination of IC_{50} values, the percent activity at each inhibitor concentration was calculated from HPLC integration data using equations 1 and 2: To determine an IC_{50} value for a given inhibitor, the plot of % activity versus [inhibitor] was fit to equation 3, with % $activity_0$ denoting hGOAT activity in the presence of the vehicle alone. All reported IC_{50} values represent the average of a minimum of three independent trials.

$$(1)\ \%\ \text{activity} = \frac{\%\ \text{peptide acylation in presence of inhibitor}}{\%\ \text{peptide acylation in absence of inhibitor}}$$

$$(2)\ \%\ \text{peptide octanoylation} = \frac{\text{Fluorescence of acylated peptide}}{\text{Total peptide fluorescence (acylated and non-acylated)}}$$

$$(3)\ \%\ \text{activity} = \%\ \text{activity}_0 * \left(1 - \frac{[\text{inhibitor}]}{[\text{inhibitor}] + \text{IC}_{50}}\right)$$

Confirmation of hGOAT expression in transfected HEK 293 cells by Western blot. hGOAT transfected cells were harvested by treatment with 0.25% trypsin-EDTA at 37°C for 5 minutes. Cells were then transferred to an Eppendorf tube and collected by centrifugation at low speed at room temperature. The media was aspirated and cells were resuspended in 1x sample buffer (0.33 M Tris HCl, pH 6.8, 0.1 M SDS, 14% glycerol, and 0.5 M DTT) and 50 mM HEPES pH 7.0 in a total volume of 45 µL. Samples were heated to 50.2 °C for 5 minutes and then incubated at room temperature for 15 min prior to gel loading.[3] Samples were loaded onto a 12 % Tris-glycine SDS-polyacrylamide gel and run at 110 V for 1.5 hrs. Each gel was loaded with a negative control (empty vector (EV) microsomal protein) and amino-terminal FLAG-BAP Fusion protein as a positive control (Millipore Sigma, P7582-100UG, 1:200 dilution, 50 µL total volume) using a previously published protocol.[1] Following SDS-polyacrylamide electrophoretic separation, proteins were transferred to a polyvinylidene difluoride (PVDF) membrane (BioRad, Trans-Blot turbo RTA transfer kit). The PVDF membrane was activated with methanol incubation for 30 seconds followed by equilibration in transfer buffer (20% v/v methanol, 48 mM Tris base, 39 mM glycine and 0.034% v/v SDS) before transfer. Proteins were transferred to the membrane for 30 minutes at 1.3 A / 25 V using a transfer kit per manufacturer's instructions. Following transfer electroblotting, the PVDF membrane was blocked for 3 hours with 5% v/v nonfat milk in TBST buffer (Tris 10 buffered saline (TBS, 0.05M Tris and 0.14M NaCl) with 0.1% v/v Tween 20). FLAG antibody (HRP-conjugated DYKDDDDK Tag Antibody, Invitrogen catalog number PA1-984B-HRP, 1:2000 dilution, 10 mL total volume) was prepared in 5% nonfat milk in TBST buffer and membrane was incubated with the antibody overnight at 4 °C. The membrane was washed with TBST (6 x 5 mL) and treated with West Pico Chemiluminescent substrate-imaging reagent

(Thermo Scientific), followed by imaging on a ChemiDoc XRS+ gel documentation system (BioRad).

Anti-MBOAT4 antibody peptide competition assay in LNCaP cells. The total IgG concentration of the anti-MBOAT4 antibody was measured on a Nanodrop spectrophotometer using the IgG setting to be 0.436 μg/μL. The competitor peptide (human GOAT residues 271-288, FGPELGQSPGEEGYVPDA) was commercially synthesized by BioBasic. The lyophilized competitor peptide was dissolved in 1 mL antibody dilution solution and concentration was measured on Nanodrop using the protein setting to be 1 μg/μL. Solutions were prepared to contain anti-MBOAT4 antibody diluted 1:250 (final concentration 1.75 μg/mL) with and without the competitor peptide, with the peptide in 1-5 fold excess relative to antibody concentration based on a mass to mass ratio. Control solutions contained the anti-MBOAT4 antibody along (1:250 dilution) solution or the 5X competitor peptide solution alone.

LNCaP cell were plated on PDL-coated glass coverslips in a 6-well plate at a density of 0.75 x 10^6 cells per well. Cells were incubated for 48 hours at 37°C and 5% CO_2. Following incubation, the coverslips were transferred cell-side up to a clean 6-well plate and rinsed three times with 1 mL 1X PBS. Cells were then fixed in 4% PFA for 30 minutes, rinsed three times with 1 mL 1X PBS, quenched with 1 mL 50 mM NH4Cl in 1X PBS for 10 minutes, then rinsed three times with 1 mL 1X PBS. Cells were permeabilized with 1 mL 0.1% Triton-X in 1X PBS for 5 minutes, rinsed five times with 1 mL 1X PBS, blocked with 2% normal donkey serum (NDS) in 1X PBS for 30 minutes, then rinsed three times with 1 mL 1X PBS.

Prior to immunostaining, primary antibody/peptide solutions were quickly spun in a mini centrifuge to remove antibody/serum complexes. Following centrifugation, 80 μL drops of

supernatant were placed on clean parafilm in the antibody-binding dark chamber. Coverslips with fixed cells were placed on the drops cell-side down and incubated at 4°C overnight in dark conditions. Coverslips were removed from the antibody/peptide solution and transferred to a clean piece of parafilm cell-side up. Cells were washed five times with 100 μL 1X PBS, 5X. the secondary antibody (goat anti-rabbit conjugated AlexaFluor 488, 1:500) was bound similarly to primary antibody, but for 1 hour at room temperature. Cells were washed five times with 100 uL 1X PBS before a final rinse in clean ultra pure water and mounting on a glass microscope slide with ProLong mounting media. Samples were cured overnight at room temperature, in dark conditions before imaging. Imaging and image analysis was performed as described in Experimental methods.

Table S1. Analytical data for peptide ligands

Ligand	Peptide Sequence	$[M+H]^+$ calcd	$[M+H]^+$ found	Purity (%)	Yield (%)
1	H-GS-Dap-FLSPY-NH_2	855.4365	855.3387	99	36
2	H-GS-(C8-Dap)-FLSPY-NH_2	981.5409	981.4415	98	20
3	H-GS-(6FN-Dap)-FLSPY-NH_2	1027.4689	1027.5400	99	25
4	H-Aib-S-Dap-FLSPY-NH_2	883.4678	883.3658	99	26
5	H-Inp-S-Dap-FLSPY-NH_2	909.4834	909.4159	99	28
9	H-GS-(C8-Dap)-FLSPE-NH_2	947.5202	947.8200	96	22
10	H-GS-(C8-Dap)-FLSPN-NH_2	932.5205	932.8500	99	25
11	H-GS-(C8-Dap)-FLSPT-NH_2	919.5253	919.8689	99	24
12	H-GS-Dap-Nal-1-LSPT-NH_2	843.4365	843.3524	99	10
13	H-GS-Dap-Nal-2-LSPT-NH_2	843.4365	843.3524	98	34
14	H-Inp-S-(6FN-Dap)-Nal-1-LSPT-NH_2	1069.5159	1069.5227	99	33
15	H-GS-Dap-Nal-1-LSPT-(SulfoCy5-K)-NH_2	$[M+H]^{2+}$ = 798.3673	$[M+H]^{2+}$ = 798.5341	95	7

Table S2. Cell imaging and scoring for GOAT transfection and ligand **15** uptake.

Trial 1

hGOAT variant	Total cells counted (n)	# Cells displaying hGOAT expression (anti-FLAG immunofluorescence)	# Cells displaying ligand 15 uptake (Cy5 fluorescence)	% of hGOAT-positive cells exhibiting ligand 15 uptake
**Wild type**	100	20	10	50
**Empty Vector**	100	0	0	N/A
**H338A**	100	13	0	N/A
**R304A**	100	26	6	23
**N307A**	100	28	8	29

Trial 2

hGOAT variant	Total cells counted (n)	# Cells displaying hGOAT expression (anti-FLAG immunofluorescence)	# Cells displaying ligand 15 uptake (Cy5 fluorescence)	% of hGOAT-positive cells exhibiting ligand 15 uptake
**Wild type**	100	21	12	57
**Empty Vector**	100	0	0	N/A
**H338A**	100	16	0	N/A
**R304A**	100	28	10	36
**N307A**	100	24	9	38

Trial 3

hGOAT variant	Total cells counted (n)	# Cells displaying hGOAT expression (anti-FLAG immunofluorescence)	# Cells displaying ligand 15 uptake (Cy5 fluorescence)	% of hGOAT-positive cells exhibiting ligand 15 uptake
**Wild type**	100	26	16	62
**Empty Vector**	100	0	0	N/A
**H338A**	100	10	0	N/A
**R304A**	100	21	8	38
**N307A**	100	22	9	41

Table S3. Plasmids used in this study.

Plasmid Name	Genotype	Source
WT hGOAT	pcDNA 3.1_Mb4.WT<br>(hGOAT-3xHA -FLAG-His_6)	This study
H338A hGOAT	pcDNA 3.1_Mb4.H338A<br>(H338A hGOAT -3xHA -FLAG-His_6)	This study
R304A hGOAT	pcDNA 3.1_Mb4.R304A<br>(R304A hGOAT -3xHA -FLAG-His_6)	This study
N307A hGOAT	pcDNA 3.1_Mb4.N307A<br>(N307A hGOAT -3xHA -FLAG-His_6)	This study

Table S4. Primers used in this study.

Primer name	Primer sequence
hGOAT H338A mutagenesis	Forward: CAGCTTGGTGGGCCGGACTGCACCCTGG
	Reverse: CCAGGGTGCAGTCCGGCCCACCAAGCTG
hGOAT R304A mutagenesis	Forward: GAATCTCGGTCTTCTCCGCTAAGTGGAACCAAAGC
	Reverse: GCTTTGGTTCCACTTAGCGGAGAAGACCGAGATTC
hGOAT N307A mutagenesis	Forward: CTCCCGTAAGTGGGCCCAAAGCACTGCTCGC
	Reverse: GCGAGCAGTGCTTTGGGCCCACTTACGGGAG

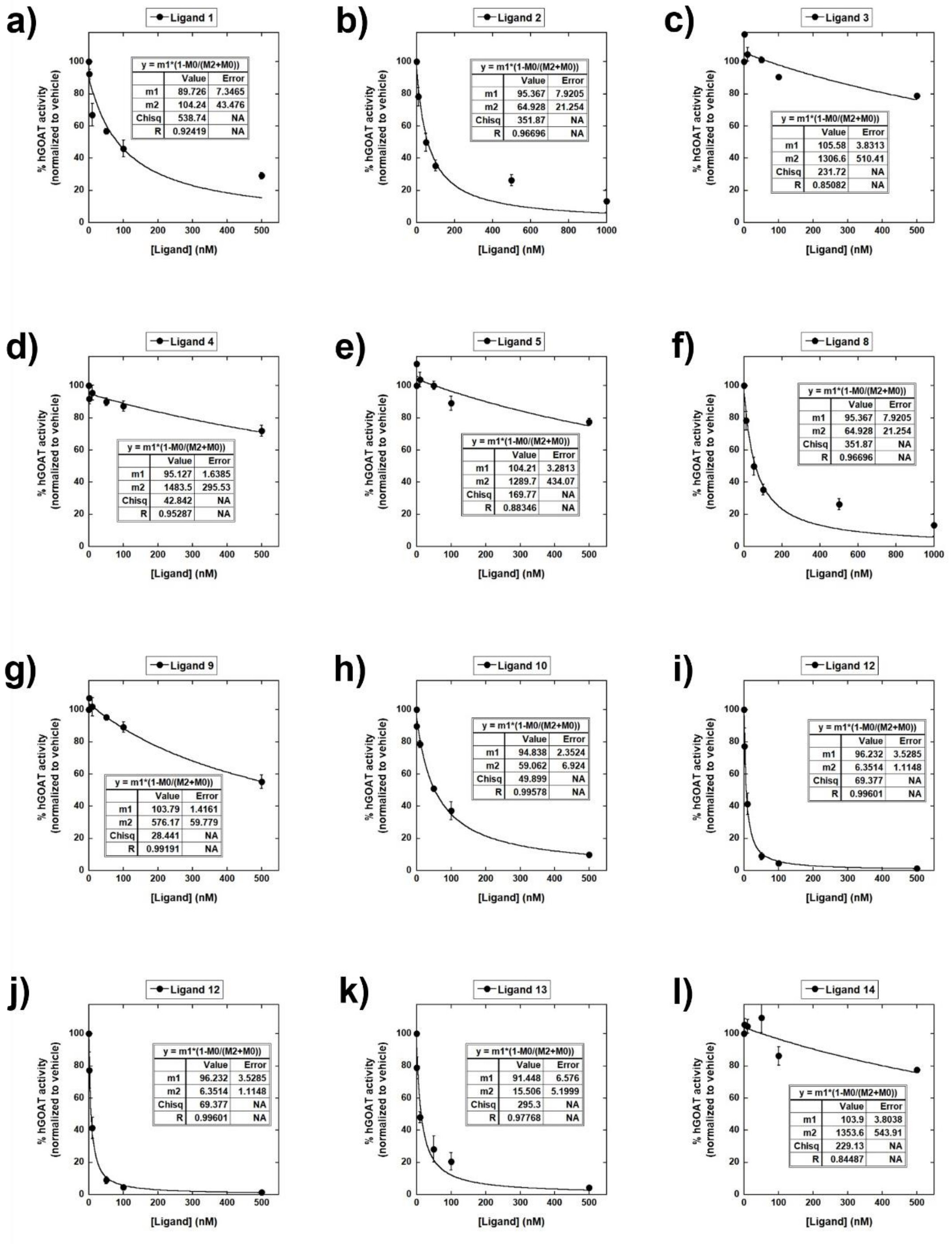

Figure S1. Dose-response curves for hGOAT inhibition by peptide ligands. All reported IC_{50} values against hGOAT represent the average of three independent trials, and error bars represent one standard deviation. Inhibition was measured and analyzed as described in Experimental Procedures.

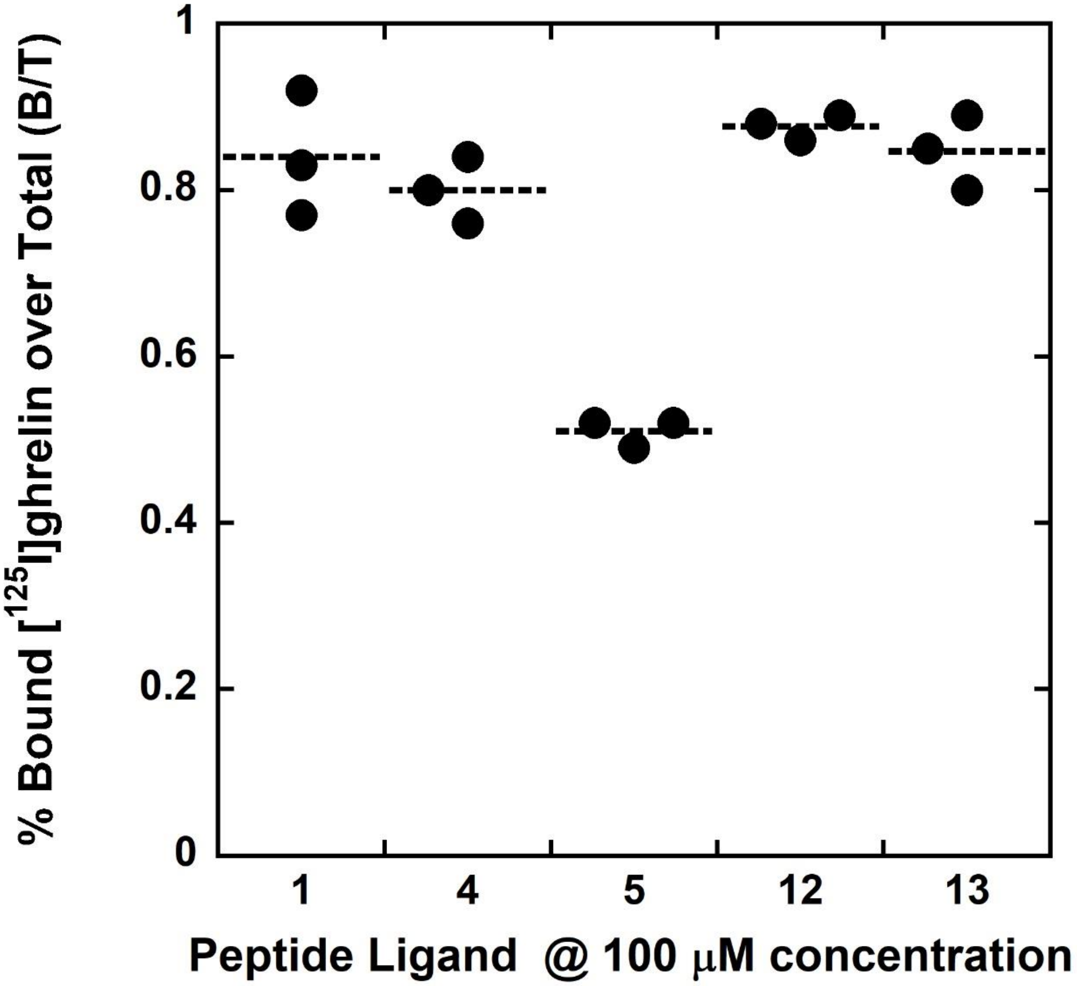

Figure S2. GHSR binding competition assays for peptide ligands. Peptide ligands 1, 4, 5, 12, and 13 were tested for binding to the GHSR receptor with a competition displacement assay using ^{125}I-labeled ghrelin as described in Supporting Methods. All ligands were tested at a concentration of 100 μM. None of the ligands induced more than a 50% displacement of ghrelin from the GHSR receptor under these conditions, yielding a lower limit of 100 μM for the IC_{50} of these peptide ligands against GHSR as reported in Table 1.

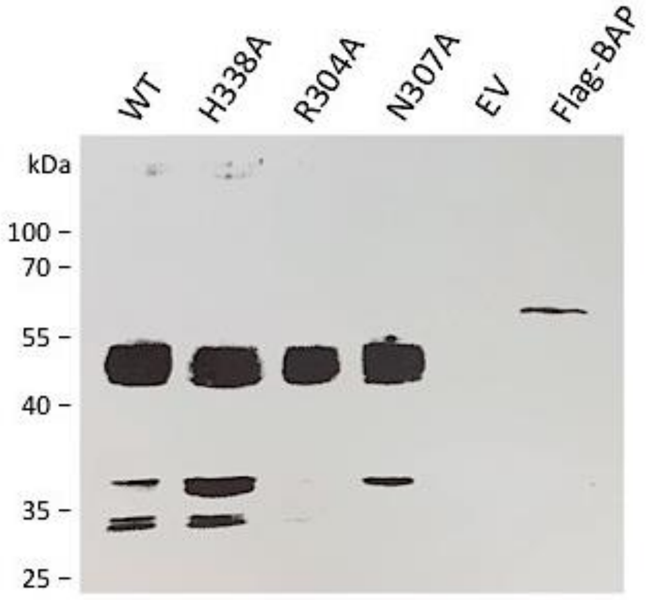

Figure S3. Western blot verification of hGOAT expression in transfected HEK 293 cells. hGOAT variant expression confirmation by anti-FLAG Western blotting with expected size of hGOAT variants (49 kDa). Western blots were performed as described in the Methods section. WT, wild type hGOAT; EV, empty vector expression; FLAG, FLAG-BAP fusion protein positive control.

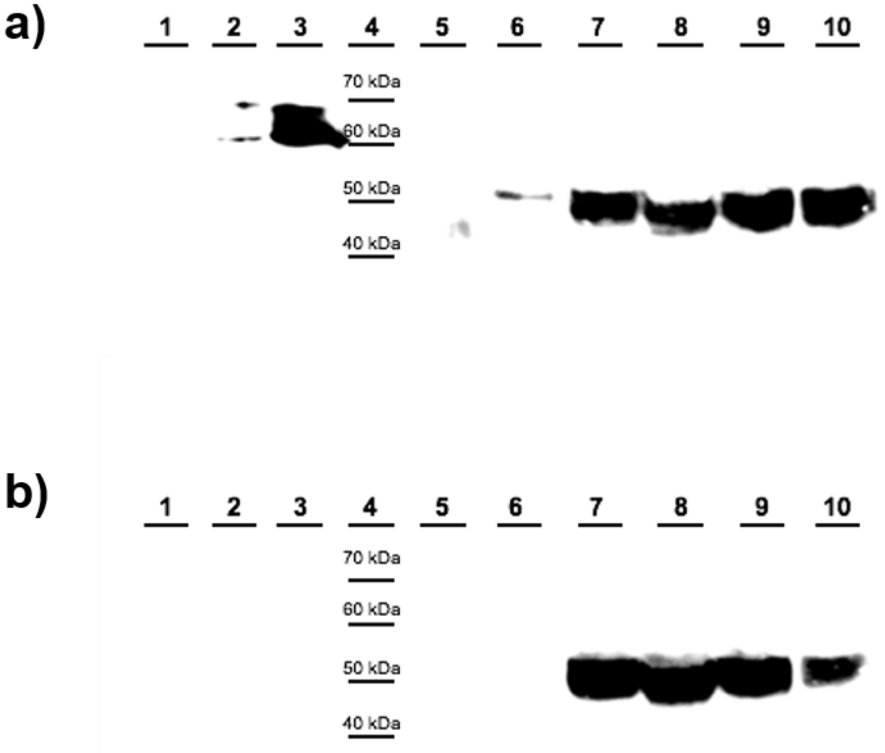

Figure S4. Western blot verification of anti-MBOAT4 antibody. Human and mouse ghrelin O-acyltransferases with a C-terminal 3xHA-Flag-His$_6$ tag were expressed in insect cells using the Bac-to-Bac baculoviral system (Invitrogen), and 30 μg total membrane protein was loaded in each well on a 10% SDS-PAGE gel. Protein was transferred to a PVDF membrane and blotted with anti-FLAG antibody (a) and anti-human MBOAT4 antibody (b). Lanes: 1, Uninfected Sf9 membrane protein fraction; 2, Empty vector infected SF9 membrane protein fraction; 3, FLAG-BAP fusion protein positive control; 4, Protein Ladder; 5, Blank lane; 6, Mouse ghrelin O-acyltransferase (mGOAT)-infected Sf9 membrane protein fraction; 7-10, Human ghrelin O-acyltransferase (hGOAT)- infected SF9 membrane protein fractions. The FLAG-tagged hGOAT was detected by both antibodies, while FLAG-tagged mGOAT is only detected by the anti-FLAG antibody consistent with the antibody specificity reported by the manufacturer (Cayman Chemical). Western blots were performed as described in the Experimental section.

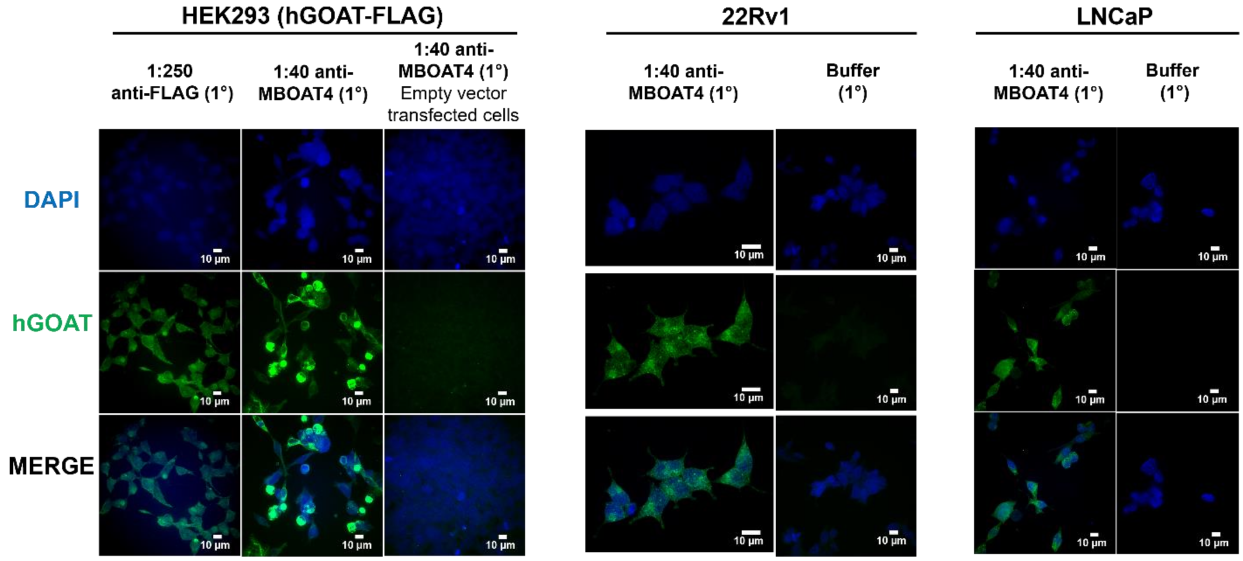

Figure S5. Immunofluorescence validation of anti-MBOAT4 antibody in HEK293, 22Rv1, and LNCaP cells. Cells were fixed and labeled with either the anti-MBOAT4 antibody or with buffer alone, followed by a fluorescently conjugated secondary antibody. In cell expressing hGOAT, immunofluorescence staining is only observed in cells labeled by the primary anti-MBOAT4 antibody. Cells were fixed, stained, and imaged as described in Experimental Procedures.

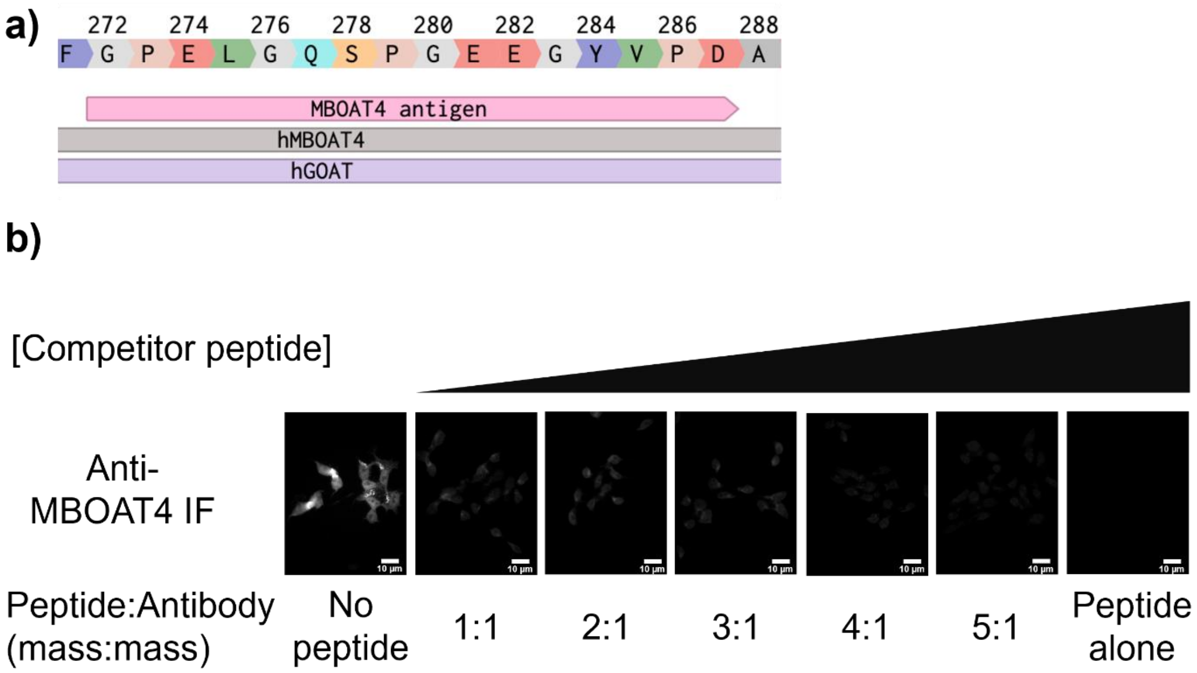

Figure S6. Loss of GOAT immunofluorescence upon peptide competition verifies epitope specificity of anti-MBOAT4 antibody. a) BLAST analysis of the immunizing antigen peptide for the anti-MBOAT4 antibody (sequence provided by Cayman Chemical) indicates the peptide sequence is unique in the human proteome. b) Dose-dependent loss of immunofluorescence signal for GOAT/MBOAT4 in LNCaP cells when the antibody is preincubated with the competitor antigen. Cells were fixed, labeled, and imaged as described in Supplemental Methods.

References

1. Charron, C. L., et al., Structure-activity study of ghrelin(1-8) resulting in high affinity fluorine-bearing ligands for the ghrelin receptor. *J Med Chem* **2017,** *60* (17), 7256-7266.

2. Darling, J. E., et al., A fluorescent peptide substrate facilitates investigation of ghrelin recognition and acylation by ghrelin o-acyltransferase. *Anal Biochem* **2013,** *437* (1), 68-76.

3. Sieburg, M. A., et al., Biochemical assays for ghrelin acylation and inhibition of ghrelin o-acyltransferase. *Methods Mol Biol* **2019,** *2009*, 227-241.

4. McGovern-Gooch, K. R., et al., Ghrelin octanoylation is completely stabilized in biological samples by alkyl fluorophosphonates. *Endocrinology* **2016,** *157* (11), 4330-4338.
